# Towards image-based cancer cell lines authentication using deep neural networks

**DOI:** 10.1038/s41598-020-76670-6

**Published:** 2020-11-16

**Authors:** Deogratias Mzurikwao, Muhammad Usman Khan, Oluwarotimi Williams Samuel, Jindrich Cinatl, Mark Wass, Martin Michaelis, Gianluca Marcelli, Chee Siang Ang

**Affiliations:** 1 School of Engineering and Digital Arts, University of Kent, Canterbury, UK; 2 Department of Computer Science, The National University of Computer and Emerging Sciences, B Block, Faisal Town, Lahore, Pakistan; 3 Shenzhen Institutes of Advanced Technology, Chinese Academy of Sciences, Shenzhen, China; 4 Institut Für Medizinische Virologie, Klinikum Der J.W. Goethe-Universität, Frankfurt am Main, Germany; 5 School of Biosciences, The University of Kent, Canterbury, UK

**Keywords:** Computational biology and bioinformatics, Machine learning

## Abstract

Although short tandem repeat (STR) analysis is available as a reliable method for the determination of the genetic origin of cell lines, the occurrence of misauthenticated cell lines remains an important issue. Reasons include the cost, effort and time associated with STR analysis. Moreover, there are currently no methods for the discrimination between isogenic cell lines (cell lines of the same genetic origin, e.g. different cell lines derived from the same organism, clonal sublines, sublines adapted to grow under certain conditions). Hence, additional complementary, ideally low-cost and low-effort methods are required that enable (1) the monitoring of cell line identity as part of the daily laboratory routine and 2) the authentication of isogenic cell lines. In this research, we automate the process of cell line identification by image-based analysis using deep convolutional neural networks. Two different convolutional neural networks models (MobileNet and InceptionResNet V2) were trained to automatically identify four parental cancer cell line (COLO 704, EFO-21, EFO-27 and UKF-NB-3) and their sublines adapted to the anti-cancer drugs cisplatin (COLO-704^r^CDDP^1000^, EFO-21^r^CDDP^2000^, EFO-27^r^CDDP^2000^) or oxaliplatin (UKF-NB-3^r^OXALI^2000^), hence resulting in an eight-class problem. Our best performing model, InceptionResNet V2, achieved an average of 0.91 F1-score on tenfold cross validation with an average area under the curve (AUC) of 0.95, for the 8-class problem. Our best model also achieved an average F1-score of 0.94 and 0.96 on the authentication through a classification process of the four parental cell lines and the respective drug-adapted cells, respectively, on a four-class problem separately. These findings provide the basis for further development of the application of deep learning for the automation of cell line authentication into a readily available easy-to-use methodology that enables routine monitoring of the identity of cell lines including isogenic cell lines. It should be noted that, this is just a proof of principal that, images can also be used as a method for authentication of cancer cell lines and not a replacement for the STR method.

## Introduction

Cancer is a major cause of death in developed countries and increasingly also in developing countries. Based on the GLOBOCAN 2018 estimates of cancer incidence and mortality by the International Agency for Research on Cancer, an estimated 18.1 million cancer cases (17.0 million excluding nonmelanoma skin cancer) were newly diagnosed in 2018, and 9.6 million individuals (9.5 million excluding nonmelanoma skin cancer) died from cancer in 2018. The cancer incidence increases with age. Hence, the number of cancer cases is anticipated to rise further as a consequence of a rising lifespan. Thus, research is needed to improve cancer therapies.

Cancer cell lines are cancer cells that have been isolated from human or animal cancers and that can be grown continuously as cell cultures in a laboratory. They are important and probably the most commonly used model system for both studying cancer biology and the discovery of novel anti-cancer drugs^1,2^. However, many cell lines are misidentified, i.e. they have been swapped or contaminated with other cell lines, and as a result, researchers may work with cells that are not what they think they are. This has been a problem since the work with cancer cell lines started and continues to be a problem today. Short Tandem Repeat (STR) analysis has been available as a reliable method to identify the genetic origin of a cancer cell line for a while^3^. Short Tandem repeats are accordion-like stretches of DNA containing core repeat units of between two and seven nucleotides in length that are tandemly repeated from approximately a half dozen to several dozen times^4^. By comparing this DNA, scientists have successfully managed to determine samples coming from the same genetic origin, and have been widely applied in forensic studies. Although STR test have been available for a while, 15–20% of the currently used cell lines have been estimated to be misidentified^5^. For example, in a study based on the analysis of 482 different human tumor cell lines, up to 96 cell lines were misidentified^6^. Moreover, another study^6^ found that STR profiling alone is insufficient to exclude inter-species cross-contamination of human cell lines, and the study argued for the need of additional testing and authentication methods. Hence, additional authentication methods that can be easily applied in the laboratory are highly desirable.

Additionally, methods are needed that reliably discriminate between isogenic cell lines, such as clonal sublines and drug-adapted cancer cell lines, since this is not achievable using STR analysis. Drug-adapted cancer cell lines are used as models of acquired drug resistance and have been used to identify many relevant drug resistance mechanisms^7–11^. We have established the Resistant Cancer Cell Line (RCCL) collection, the worldwide largest collection of drug-adapted cancer cell lines, currently consisting of > 1,500 models of acquired resistance^12^. Automated image recognition approaches may be an additional way to authenticate cells that may complement STR analysis and enable the differentiation between isogenic cell lines^13^.

Conventional machine learning models such as Support Vector Machines (SVM), Artificial Neural networks (ANN), Naïve Bayes and Decision Trees, have been used to perform cancer cell classification based on microscopy images. These models have been able to detect similarities/differences between cancer cell images, which can be missed by the human eye. Nusaibah et al.^14^ used decision tree algorithms to perform classification of cancer cells into benign and malignant with a precision of 97.7%. In another study, Smita et al.^15^, found that SVM performed best out of a range of conventional machine learning models at classifying cancer images. The use of conventional machine learning to perform classification can be complex, relying on manual feature extraction (feature engineering) from images, which can be challenging^13^. The performance of most conventional machine learning methods are highly dependent on feature engineering^16^. Therefore, some researchers have turned their attention to deep learning approaches, which can extract and organize the discriminative features from data without the need of manual feature engineering. Deep learning approaches have achieved remarkable performance in tasks such as speech recognition^17^, natural language processing^18^, and image classification^19^. Deep learning has begun to be used for image recognition in cancer, for example, Angel et al.^20^ and Jinhua et al.^21^ both used convolutional Neural Networks (CNN) for detecting the presence of invasive tumour cells in breast cancer tissue samples.

However, the performance of deep learning largely depends on the amount of training data available. Training a deep learning model is computationally expensive and time-consuming^22^. When learning on a limited amount of data, data augmentation and transfer learning are two methods that can be used to improve the performance of deep learning models. Data augmentation is a technique which is used to artificially increase the diversity and size of the training sample. This is done by performing operations like shift, zoom, flip and rotation on the available training data. Zeshan at.al^23^ reported how data augmentation contributes to the performance of deep learning models in classification of medical images. Authors further analysed which data augmentation techniques retain the properties of the original image as it contributes to the model performance. Another study has surveyed the significance of different data augmentation techniques in fields which is difficult to get enough training data sets^24^.

Transfer learning involves the use of pre-trained models designed to perform a particular task in one context, their top layers are removed and new layers attached, to be trained to perform a new task, in a different context. Since only the last few layers of the model will be retrained, computation costs and training time are reduced and only a smaller amount of data is needed. There are several pre-trained models available, such as GoogLeNet^25^, VGG^26^, MobileNet^27^, and InceptionResNet V2^28^. Most of the existing pre-trained models for image classification are trained on the Large-Scale Visual Recognition Challenge (ILSVRC) ImageNet dataset^29^, a general object recognition dataset which includes 1000 classes^30^. Transfer learning technique was used to classify lung cancer types in a study by Nicolas et al.^31^ with a high level of accuracy (0.97 AUC- Area under the Receiver Operating Characteristic Curve). Using Inception V3, a pre-trained model on ImageNet, it was also possible to predict the ten most mutated genes in lung adenocarcinoma (LUAD)^32^, using the same model^31^. Sung-Jin et al.^33^ used VGG19, another pre-trained model on ImageNet, to perform classification in molecular diagnostics, a collection of techniques used to analyse biological markers in the genome and proteome. Mark et al.^22^ explored several deep learning architectures to detect glaucomatous optic neuropathy in fundus photography with Transfer learning, outperforming all other methods. J. Huang et al.^34^ proposed InceptionResNet V2 and MobileNet as a feature extractor together with Faster Regions Convolutional Neural network (R-CNN) as a meta-architecture for the image identification and detection. InceptionResNet V2 has provided a good accuracy at the cost of more computational time, whereas MobileNet’s accuracy is comparable to the VGG model but has 1/30 of the computational cost. Google’s Inception V3, a deep learning model pre-trained on ImageNet, was used to perform the classification of histopathological images of breast cancer in a study by Jongwon et al.^35^.

In the laboratory environment, microscopic images are the most digitised form of images which can be produced from different samples under investigation. Deep learning and its techniques have been playing an important role in computerised analysis of these microscopic images. For example, Oei et.al^36^ trained a CNN to classify between normal and breast cancer cells using actin-labelled fluorescence microscopy images based on the knowledge that the actin cytoskeletons differ between normal tissue and tumour tissue, while others have used inverted phase microscopy images to classify breast cancer cell lines from normal breast cell lines^37^. Most recently, Meng et al.^38^ used a CNN trained on cell images of single cells from ultra-high-throughput microscopy (asymmetric detection time-stretch optical microscopy) to classify four different cell types with > 99% accuracy. However, they used two epithelial breast cancer cell lines (MCF-7, MDA-MB-231), one acute myeloid leukaemia cell line (THP-1), and peripheral blood mononuclear cells (PBMCs).

In this study, we investigate the use of two deep learning models, MobileNet and InceptionResNetV2, in the authentication of cancer cell lines through a classification process by using the microscopic images of the cancer cell lines. Both models are based on convolutional neural networks (CNN), which have the capabilities of finding their own related features from the training data without the need of feature engineering. InceptionResNet combines the two major concepts of Inception and ResNet models as used by C. Szegedy et al.^28^. Inception incorporates each convolution in parallel and concatenates them at the end as proposed by C. Szegedy et al.^27^. The key benefit of the architecture is that, it increases the number of units at each stage without increasing the computational complexity much^28^. Each inception unit has several non-linear convolution modules with various resolution which makes it more applicable to tasks that need to process data with multiple resolutions like medical images^33^. As Inception networks tends to be very deep, and residual connections adopted from ResNet perform good in training deep networks, the filter concatenation stage of the Inception networks can be replaced by residual connections^28^ and give rise to InceptionResNet which takes advantage of both Inception and Residual architectures. Another model, MobileNet, deploys Depthwise Separable Convolution which applies a single filter to each input channel instead of standard convolution^29^. In MobileNet, a standard convolution is factorised into Depthwise convolution and a 1 × 1 convolution called a pointwise convolution. Depthwise convolution applies a single filter to each input channel. The pointwise convolution then applies a 1 × 1 convolution to combine the outputs of the Depthwise convolution. The MobileNet reduces the computational costs by avoiding standard convolution which filters and combines inputs into a new set of outputs in one step^29^.

The cell line data we used contain a set of four parental cancer cell lines and their sublines adapted to anti-cancer drugs. This dataset enables us to consider the ability to distinguish between cancer cell lines of different genetic origins and also those of the same genetic origin. We further perform separate classifications of the four parental cell lines and their respective drug-adapted sublines in a separate classification task as part of the authentication process; this is a task which cannot be performed by STR authentication. In contrast to Meng et al.^38^, whose method depends on expensive machinery that is only available in very few specialised laboratories, our approach is based on phase-contrast images from well plates or cell culture flasks that can be obtained with every standard inverted cell culture microscope and will, therefore, be available to every standard cell culture lab. Our authentication method for drug-adapted sublines based on deep learning will complement STR and help to ensure that researchers know what cell lines they are working with.

## Materials and methods

Two deep learning models were explored for the analysis, InceptionResNet V2 and MobileNet^34^. For model training, random initialisation of the model weights (training from scratch) and transfer learning methods were used and compared. In our analysis, for transfer learning, we used weights of the models pre-trained on ImageNet, a large-scale dataset used for Large-Scale Visual Recognition Challenge (ILSVRC), which contains 1.2 million images of general objects^29^. Several strategies were tested for data pre-processing and training in order to find the optimum strategy and the optimum model configuration for our problem (see Table 2). The tests used to find the optimum training strategy and the optimum model were conducted through a pilot classification task as explained in “Pilot classification task” section of materials and methods of this paper. In “Datasets” section, the datasets are introduced and described. Data pre-processing is summarised in “Data pre-processing” section. “Performance measure metric” section explains the metric used as a performance measure of models during our experiments. “Hyper-parameters tuning of the optimal model” section presents further hyper-parameter tuning of the optimum model obtained from the pilot classification task in “Pilot classification task” section. Using our optimal model found from pilot classification in “Pilot classification task” section, which was further fine-tuned in “Hyper-parameters tuning of the optimal model” section, we conducted several authentication tasks which are covered in “Authentication using the optimal model” section of this paper.

### Datasets

Two datasets were used, the cancer cell lines dataset, which is the objective of our authentication task, and the breast cancer cells dataset, which is made of publicly available^36^ breast cancer cell images. For transfer learning, the two models used, MobileNet and InceptionResNet V2, were pre-trained on non-medical images, the ImageNet^30^. After determining and fine tuning the hyper-parameters of the optimum model for our problem, a multi stage transfer learning approach was therefore conducted on the optimum model configurations following pilot classification, by using the breast cancer cells dataset. The breast cancer cell images were used as an intermediate transfer learning stage before training the model on our target task of cancer cell lines dataset.

#### Cancer cell lines dataset

**Figure 1:**
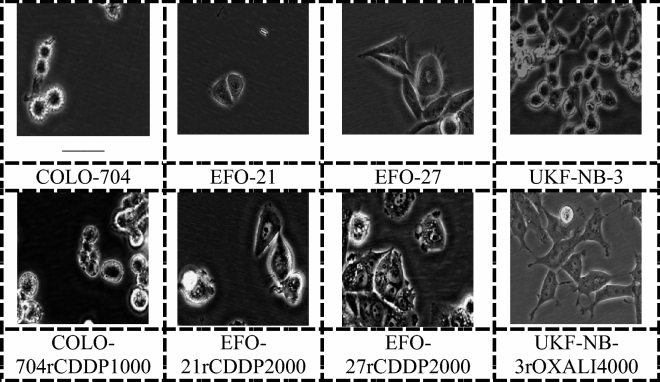
Cancer cell lines sample images.

The dataset consisted of microscopy images of parental cancer cell lines and their sublines, which had been adapted to grow in the presence of anti-cancer drugs. The set of cancer cell lines consisted of three ovarian cancer cell lines (EFO-21, EFO-27, COLO704) and their sublines adapted to the anti-cancer drug cisplatin, as well as the neuroblastoma cell line UKF-NB-3 and its subline adapted to the anti-cancer drug oxaliplatin. The ovarian cancer cell lines were obtained from DSMZ (Braunschweig, Germany). The neuroblastoma cell line was established from a bone metastasis of a stage IV neuroblastoma patient^39^, in accordance with relevant guidelines and regulations. All drug-resistant sublines had been established by continuous exposure to stepwise increasing drug concentrations as previously described^12,39^ and were derived from the Resistant Cancer Cell Line (RCCL) collection^7^. The cisplatin-resistant ovarian cancer sublines had been adapted to 1 µg/mL (COLO-704^r^CDDP^1000^) or 2 µg/ml (EFO-27^r^CDDP^2000^ and EFO-21^r^CDDP^2000^) cisplatin. The oxaliplatin-resistant UKF-NB-3 subline (UKF-NB-3^r^OXALI^4000^) was adapted to 4 µg/mL oxaliplatin^39^. Table 1 shows the number of images per cell line. Image samples for each class are shown in Fig. 1.

**Table 1:** Number of images per cell line.

Cell line	Number of images (n)
COLO-704	220
COLO-704rCDDP1000	270
EFO-21	220
EFO-21rCDDP2000	220
EFO-27	220
EFO-27rCDDP2000	220
UKF-NB-3	201
UKF-NB-3rOXALI4000	170

This dataset was collected at the School of Bioscience, University of Kent, Canterbury Campus, UK. All the images are in RGB, JPEG (Joint Photographic Experts Group) format with a resolution of 2560 × 1922 pixels.

#### Breast cancer cells dataset

As most of the available pre-trained models for transfer learning are trained on non-medical images, we proposed a multi-stage transfer learning through an inter-mediate training step using breast cancer cell images, to make sure our selected model had the knowledge of medical images before fine-tuning it for our cancer cell lines cell line authentication task. A multi-stage transfer learning is a technique of transfer learning in which a model can undergo several transfer learning before fine-tuned to its target task. Mostly, the intermediate transfer learning stage involves training a model with a data set from the domain similar to domain of the target task. For this multi-stage transfer learning, we used publicly available breast cancer cells data set. The data had been produced by the Laboratory of Vision, Robotics and Imaging (VRI) at the Federal University of Parana, and collected from 82 patients and comprised of 7909 microscopy images of breast tumour tissue. It is divided into two categories with 5429 malignant samples and 2480 benign samples, both with 700 × 460 pixels resolution and 3 channel RGB.

### Data pre-processing

Prior to model training, pre-processing steps like image splitting, cross validation, conversion to grayscale, data normalisation^40^ and different data augmentation methods were performed^24,41^. Data normalisation is a technique of restricting features of the data to within a specific range^42^. In machine learning, the technique ensures each parameter (pixels in our case) has similar data distribution. Data normalisation was performed to remove variance in brightness and to enhance the contrast of the images. This was done by subtracting each image from its mean pixel-intensity value to make convergence faster while training the network. In the case of RGB images, the mean was calculated channel wise. All the processing conducted in this paper was done only on the cancer cell line dataset which is our target task. Our models were trained with both grayscale and RGB formats for comparison; the conversion to grayscale, data normalisation and augmentation were done online during the training process. For image splitting, the dataset was separated into two main categories, parental cancer cell lines and their drug-adapted sublines. The four parental cancer cell lines, their four sublines of drug-adapted and the combination of the two which form a group of eight cell lines were treated as three separate authentication problems. tenfold cross validation was performed on all three tasks. Each fold had 10% of the data set selected randomly and without repetition. As we had a relatively small data set, data augmentation approaches were used to artificially increase the sample size. As different data augmentation techniques have been proved to contribute different performance on the model^24^, two data augmentation approaches were employed and compared, which are nearest width shift and Constant width shift. Examples of augmented images are shown in the supplementary information, in Fig. S1.

### Pilot classification task

**Figure 2:**
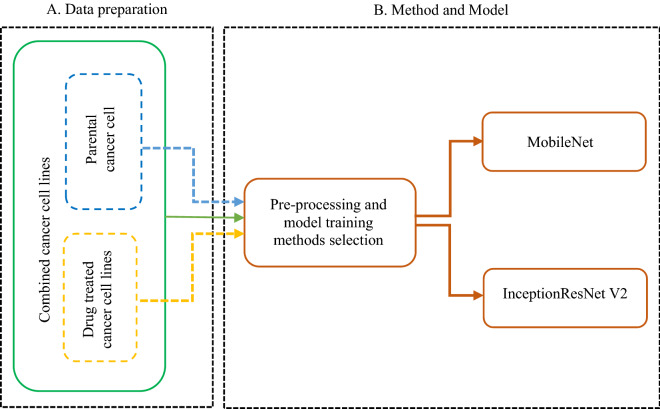
Pilot classification.

A pilot classification was conducted to determine the optimal model and training strategy for the cell lines authentication task. In this process, three authentication tasks were performed by using the two selected models with different method combinations. The three tasks were (a) the authentication of parental cancer cell lines, (b) the authentication of drug treated cancer cell lines and (c) the authentication of the combined dataset of parental and drug treated cancer cell lines (see inset A of Fig. 2). During the pilot classification, for each class in all three tasks, nine folds were used for training and validation, and the remaining fold for testing. For fair comparison across all three authentication tasks, the folds selected for training, validation and testing were kept constant during the pilot classification task. All results were analysed and compared and the optimal model and the optimal training strategy were selected based on the model’s performance. For validation and testing, only the original (un-augmented) images were used^43^. The data preparation procedures, training and model selection procedures are illustrated in insets A and B of Fig. 2, respectively. The results of this pilot classification task are shown in “Pilot classification” section.

#### Training strategies

Different strategies for data pre-processing and model training were tested to determine the optimum performing training strategy. Each possible combination of the training strategies was systematically fed to each of the two models separately and the model performance was used to determine the best performing strategy. Table 2 shows the different combinations of training strategies.

**Table 2:** Training strategies selection.

Grayscale	RGB	Without data augmentation	With data augmentation	Without transfer learning	With transfer learning	Combination number
✓		✓		✓		I
✓			Nearest	✓		II
✓			Constant	✓		III
	✓	✓			✓	IV
	✓		Nearest		✓	V
	✓		Constant		✓	VI
	✓	✓		✓		VII
	✓		Nearest	✓		VIII
	✓		Constant	✓		IX

No tests were done on transfer learning for grayscale image, because the pre-trained models used for transfer learning are trained on ImageNet dataset which has only RGB images.

#### Model selection

Two off-the-shelf Deep Neural Network models, InceptionResNet V2 and MobileNet, which have proven to work well in recognising pictures of general objects. The two models were training with different strategies to find the optimal strategy and the optimal model. Figure 2 below shows the whole process of pilot classification.

### Performance measure metric

The F1-score was chosen as a measure of performance as it considers both recall and precision. Recall is a true positive rate of a model and precision is the positive predictive value of the model. Recall and precision can be calculated as in Eqs. (1) and (2) respectively, and F1 score as in Eq. (3). TP stands for true positive, FN for false negative and FP for false positive.

1 $$ Recall = \frac{TP}{{TP + FN}} $$

2 $$ Precision = \frac{TP}{{TP + FP}} $$

3 $$ F1 = \frac{2}{{\frac{1}{recall} + \frac{1}{precision}}} $$

### Hyper-parameters tuning of the optimal model

After selecting optimal training strategies and the optimal model for our task through a pilot classification task, as explained in “Training strategies” and “Model selection” sections, further tuning of hyper-parameters was performed to find the optimal hype-parameters which can increase the performance of our optimal model. For hyper-parameters tuning of our selected model, an eight class authentication task was used as it is a more complex authentication task and therefore ideal for the fine-tuning task. The fine tuning was done by using a single fold, as in the pilot task, by keeping constant the training, validation and the test folds throughout the process. The following fine-tuning tests were conducted on our selected model;

#### Resizing and cropping

The original images in the dataset have a resolution of 2560 × 1922 pixels (RGB). In the experiments performed in the pilot classification, the images were resized by squashing the image to 299 × 299 pixels as a predefined input shape for InceptionResNetV2 and 224 × 224 pixels for MobileNet. As the predefined input shape of our best performing model was 299 × 299 pixels, several tests to transform the input image into the required input shape from our sample images were performed and compared. Govindaiah et al.^44^ resized the images in their data set to a reference image before centre cropping when training their model for screening and assessment of age-related macular degeneration from fundus images. Several resizing and cropping dimensions were tested to find an optimal approach to our problem.

To maintain the aspect ratio, the following methods were applied and then compared with image squashing and reported.

- Centre crop the image without resizing.
- Resize the image to 1280 × 961 pixels which is half of the resolution of the original image and then centre crop the image.
- Resize the image to 640 × 480 pixels which is a quarter of the resolution of the original image and then centre crop the image.
- Resize the image to 426 × 320 pixels which is 1/6 of the resolution of the original image and then centre crop the image.

#### Fully connected layers

In deep learning models, layers closer to the input learn general features of the input data while deeper layers closer to the output learn more specific features for the target problem^45^. Hence, determining the number of layers to be added on a pretrained model for transfer learning becomes an important aspect. In fact, the added layers play the crucial role in the learning of specific features for the target problem by the model. When using transfer learning, it is essential to control the depth at which the amount of ‘knowledge’ transfer between the source and target problem is optimal for the target problem. In deep neural networks, most of the parameters are in the fully connected layers^46^. Furthermore, different popular models which has won ImageNet competition has used different number of fully connected layers, AlexNet has 3 fully connected layers, GoogLeNet and ResNet which both contributed to the development of our best performing model as they use Inception and ResNet techniques, respectively, both have one fully connected layer^25,47^. We performed an experiment to find the optimal number of fully connected layers to be added and the number of neurons in those layers for our problem.

#### Batch size

The batch size, which is the number of samples processed before the model is updated during training, has an influence on the model performance in terms of both accuracy and training time. It is reported that greater the batch size the higher the accuracy of the model^48^. The idea is contradicted in^49^ where it is stated that models trained with large batch size have high chance to generalize more poorly than those trained with small batch sizes. Studies^50,51^ suggested a batch size of no more than 64 samples. In addition, a large batch size has high computational cost when training a deep learning model; previous studies^52,53^, recommended large batch size when training with large datasets, but this choice comes with optimization difficulties. Since there is no clear recommendation of the batch size to use in general, different batch sizes were tested to find the optimum batch size value for our problem.

#### Multi-stage transfer learning

A common practice in deep learning is that training and testing data should come from the same domain. In some scenarios, such as training a deep learning model with medical images, that practice becomes more challenging as it is hard to obtain sufficient medical images. Transfer learning could provide a solution by training the models with easily obtained data from a different problem and fine tune the model with the smaller dataset for the target problem. Transfer learning makes sense when low-level features from the problem you are transferring from could be helpful and are relevant to the problem you are transferring to^54^. The used models in our case were trained with ImageNet, a non-medical image dataset. To make sense of transfer learning, a multi stage transfer learning was considered by training the model through medical images, before fine tuning it on our targeted task. Some investigations into medical images have adopted multi-stage transfer learning by fine-tuning their models through other publicly available medical data sets, before fine tuning the model to their target problem. Ravi et al.^45^ observed an increment of 4 percent in F1-score when performing multi-stage transfer learning by using publicly available breast cancer data set to fine tune a deep learning model as an intermediate transfer learning stage, before fine tuning the model for their target task of classification of breast cancer. As there are common high-level features in cancer cells such as shape and size^55^, we used the breast cancer cells data set as an intermediate transfer learning stage, since there is no public available data set to our specific problem (cell lines authentication), to the best of our knowledge.

### Authentication using the optimal model

Unlike in the pilot classification task and in the fine-tuning of the optimal model were the training, validation and test folds were fixed, at this stage, nine folds were used for training and validation and one for testing for all the authentications conducted by using the optimal fine-tuned model using the optimal training strategy. The process was repeated 10 times for each fold to be used for training, validation and testing, the mean results with their standard deviation of all 10 folds are reported in this paper. In addition, we investigated the effect of sample size on cancer cell line authentication and the results are reported in this paper. The predictions of any trained deep learning model are subjected to a degree of uncertainty; a trained model can perform well in identifying some classes and perform poorly on other classes. It is necessary to understand how a trained model performs in identifying individual classes trained on instead of just an overall performance, as some classes are hard to authenticate compared to others. We studied by investigating the confidence of our optimal model in authentication of each cancer cell line separately and the findings reported. All the experiments conducted using our optimal model are as explained bellow;

#### Authentication stages

To complement the current authentication methods used in biology research (i.e. STR), which is not able to differentiate between parental cancer cell lines and their drug-adapted sublines, eight-class authentication of the mixed four (4) parental and the four (4) drug treated cancer cell lines was conducted. To apply STR profiling for authentication, standardized protocol and a data-driven, quality-controlled and publicly searchable database will be required which is a complex and time-consuming process. To complement STR with a cheaper and quicker computerised method of authenticating cancer cell lines, we further performed authentication of the four (4) parental cancer cell lines and the authentication of the four (4) drug-resistant sublines separately using our optimal fine-tuned model. Furthermore, we perform a two-class task to authenticate the parental cell lines and their drug-adapted sublines in one approach.

#### Effects of sample size

Deep learning can easily overfit when trained on a small sample size, hence it is important to study the effect of sample size when training deep learning models to avoid overfitting^13,56,57^. To study the effect of sample size, we studied our selected optimal model performance at different training sample sizes. The number of images in the test fold were kept constant while the number of images in the training folds were reduced in steps of 20%.

#### Classification confidence

Despite the high accuracy attained by a trained CNN model, it can have difficulties in predicting some of the classes^58^. This might be due to different reasons like the quality of data of that particular class or the confusion of the model due to similarities existing between the classes with low confidence. This rises the importance of studying the confidence of a trained model in prediction. I Cortés-Ciriano *et.al*^59^, studied about model confidence in assessing prediction uncertainties of their trained deep learning model for drug discovery application. To study the model’s confidence, some studies advise not to use prediction probabilities as the confidence of a deep learning model^60^. Researchers in^61^ and^62^ instead, used the probabilities of classes generated by the trained CNN models as the confidence score to reject noises and keep the predictions with high class probabilities in a face detection task. Also, in an object detection network by Redmon *et.al*^58^, probabilities of the bounding boxes containing objects were used as the confidence score of the model. We therefore studied the prediction probabilities of our model in predicting a small set of randomly selected samples from our test set by extracting their probabilities of the assigned class. The predicted probabilities were extracted from the softmax layer of the trained model. Post processing of the softmax layer has proved to be useful in delivering calibrated class probabilities in classification problems^63,64^. Both probabilities in correct and wrong predictions were studied and reported in this paper.

## Results

We adopted a *k*-fold cross validation (*k* = 10 in our case) because when comparing model’s performance using simple train/test split, different results may be obtained each time when different data samples are selected in the train/test sets^49^. It is more computationally expensive to train a model with RGB than with grayscale images. This is because RGB images carries more information which can be essential for a model to learn. Hence the two ways training with grayscale and with RGB images were tested and results compared. Both models were trained with normalised images. For data augmentation several hyper-parameters were selected. The hyper-parameters selected for augmentation reflected that the cells can be anywhere in an image independently of how the cell culture vessel is placed under the microscope. The selected method and the optimal training strategy were determined during the pilot classification task, which were later fine-tuned. InceptionResNet V2 was found to be the optimal model for our problem over MobileNet through the pilot classification task, using RGB images, nearest width shift data augmentation with transfer learning techniques (Method combination V). In fine-tuning of the optimal model, we also found that, distorting the aspect ratio by resizing the input image direct to the required input shape does not have an effect on the model performance in our problem. Multi-stage transfer learning had little impact on our problem, this might be due the kind of data we used to fine tune our model in a multi-stage process, breast cancer cells dataset, being different from our target task (cancer cell lines) data set as the breast cancer cells images were produced from the stained biopsies which are highly associated with colour changes while our cancer cell lines data set were produced from cell culture grown in the lab. Our optimal model was trained to perform different authentication tasks to automate the standard existing authentication method, the STR. Further authentication tasks were conducted using the optimal model which cannot be done by the standard authentication method, the STR, and achieved remarkable results. The following subsections presents our results.

### Pilot classification

Pilot classification was conducted on three main authentication tasks, which are the authentication of the four classes of parental cancer cell lines, four classes of their drug-adapted sublines and an eight-class combination of the previous two. This was done by fixing the training, validation and test folds through all the three tasks to determine the optimal model with the optimal method combination.

Figures S2, S3 and S4, in the supplementary document, shows that both models, InceptionResNet V2 and MobileNet, under different training strategy perform reasonably well in our pilot classification task for cancer cell line authentication, when trained on RGB images in all three authentication tasks. This is because RGB images contain a large amount of morphological information and thus play significant role in differentiating cancer cell lines^40^. As the RGB images contain more information, this makes them computational expensive to train compared to grayscale images. Both data augmentation techniques, Nearest width shift and Constant width shift, had a positive impact by significantly increasing the F1-score in both models, with nearest width shift performing better than constant width shift. With reference to training strategies selections in Table 2 in the materials and methods section of this paper, Figs. S2, S3 and S4 show that training strategy I, IV and VII, which did not include augmentation, performed worse than those which included data augmentation, i.e. training strategy II, III, V, VI, VIII and IX. Both models required more computational time for optimisation when applying augmentation since more data were generated by augmenting the training data. Transfer learning (using ImageNet) had a significant positive effect on model performance, as it always performed better (training strategy IV-V) compared to when the models were trained from scratch (training strategies I, II, III, VII, VIII, IX). With transfer learning, both deep neural network models performed better when transfer learning was combined with data augmentation (V, VI) than without data augmentation, method combination IV. A small difference in training time was observed when training the models from scratch or with transfer learning. This is because in both cases, the fully connected layers are the ones which gets trained and are the ones containing more parameters to be trained, while the deeper layers are responsible for feature extraction. MobileNet optimises faster than InceptionResNet V2 due to its size, (Figs. S2, S3 and S4). By observing Figs. S2, S3 and S4, it is clear that in both models, training with RGB and data augmentation (nearest width fill), which is described as training strategy V, performed better than any other combination of approaches.

**Figure 3:**
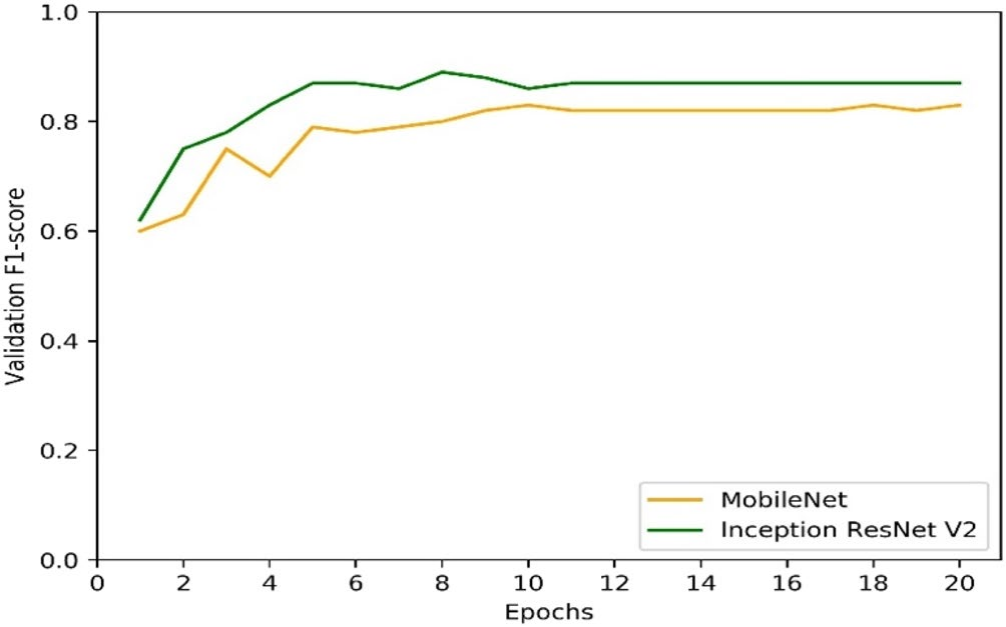
Learning curves for model comparisons.

The performance of the models (F1-scores), with the optimal strategy (method combination V) observed in our pilot classification on the authentication of an eight-class authentication task, is shown in Table 3 below. In training a deep learning model which often takes long time to train, it is essential to observe and validate the model during training to avoid overfitting. Figure 3 plots validation curves of our two models during training. From Fig. 3, it can be seen that both models generalised well on the training set, with InceptionResNet V2 performing better than the MobileNet. InceptionResNet has over 50 million trainable parameters hence become computationally expensive to train and to use the trained model for different applications compared to MobileNet which has only around 4 million trainable parameters which can even be deployed in mobile applications This may mean, MobileNet can still be considered over InceptionResNet when accuracy has to be traded over computational costs and portability. The F1-scores on the test set in Table 3 show that the trained models were able to perform well on the authentication of the unseen data. Table 3 and Fig. 3 show that InceptionResNet V2 performed better on the pilot classification task on the best method combinations determined from Table 2. The results shown in Table 3 and Fig. 3 are based on the eight classes authentication task during the pilot classification task. It took around six (6) hours to train our optimal model by following the optimal pre-processing techniques selected from the pilot classification task.

**Table 3:** Model comparison.

Model	Test F1-score
InceptionResNet V2	0.88
MobileNet	0.82

Based on results from the three authentication tasks, InceptionResNet V2 was selected as the optimum performing model compared to MobileNet. Also, a combination of RGB images, data augmentation and transfer learning (training strategy V) was observed to be the optimal configuration.

### Optimal model hyper-parameters tuning results

As described in the “Hyper-parameters tuning of the optimal model” section above of hyper-parameters tuning of the optimal model in the materials and methods section of this paper, different hyper-parameters of the optimal model were tuned to obtain the optimal performing hyper-parameters. Tuning of the optimal model was conducted by training the optimal model on by using the optimal training strategy as shown in “Pilot classification” section of results and previously described in “Pilot classification task” section for pilot classification task in the materials and methods section. The aspect ratio of the original image is 01.33:1 whereas the resized images have a 1:1 aspect ratio. Hence, resizing the image by squashing, distorts the aspect ratio of the image. It was observed that resizing only without cropping performed better, which means that the distortion of the aspect ratio did not have impact on cancer cell authentication. Resized and cropped samples and results of the model performance are shown in Fig. S5 and Table S1 respectively in the supplementary information. By visualising Fig. S5 it can be seen that, resizing and cropping loses some details of the original image while the resized image looks like the original image. Hence, resizing only was taken as the best option. For batch size selection, the results for the model learning curves are shown in Fig. S6, and final results of the model performance at different batch sizes are shown in Table S2 in supplementary information. Based on these results, one can conclude that batch size 16 and Batch size 32 had the same and highest F1 score, in comparison with the other batch sizes. However, the training accuracy and F1-score on the training set was not the same. Batch size 16 had the higher training accuracy and F1-score compared to the batch size 32. Hence, based upon the result, batch size of 16 was chosen. Different number of fully connected layers and number of neurons on the added layers were tested as shown in Table S3 in the supplementary information. An architecture with two fully connected layers had low training accuracy and F1-score, but it has a similar F1-score value as a one-fully-connected-layer architecture. Hence, we chose an architecture with one fully connected layer as it is less expensive computationally.

### Multi-stage transfer learning

A modest increment of 1 percent was observed on the F1-score when a multi-stage transfer learning was conducted compared to the single stage transfer learning. This may mean that, in multi-stage transfer learning, our optimal model was able to learn extra cancer related features from the breast cancer cells data set. This increment of F1-score produced by multi stage transfer learning might have been small because we used images of breast cancer in the intermediate stage which is little different from our target task, Unlike a study by Ravi et.al^45^ in which the intermediate stage dataset was of the same domain as the target task. Our target task, in fact, includes images from ovarian and neuroblastoma cancer cells, although they are all digital images of cancer cells. The small increment of F1-score in our case means there might be some little common features among cancer cells from different types of cancer.

### Authentication results

This section presents results of the optimal model, following hyper-parameter tuning, which are based on tenfold cross validation. With our fine-tuned optimal model and training strategy of 0.96 F1-score on authentication of four classes of parental cancer cell lines and an average of 0.91 F1-score on the four classes of drug-adapted sublines. An average of 0.91 F1-score was obtained on the eight-class authentication task. We also obtained an average of 0.95 F1-score in two-classes for authentication between the parental and its drug treated subline of the same cell line. These results are presented and further explained in the following subsections.

#### Four-class authentication task

Using the fine-tuned optimal model and training strategies, our model performed better on authentication of parental cancer cell lines than on their drug-adapted sublines (Table 4) as two separate authentication tasks. A total of 101 images in the drug-adapted cell lines were misclassified compared to just 30 images in the parental cancer cell lines. The misclassified drug-adapted were classified as other drug-adapted cell lines within the four available drug-adapted cancer cell lines while the misclassified parental cancer cell lines were classified as other parental cancer cell lines among the four available parental cancer lines as can be seen in Figs. S8 and S9 of the confusion matrices of the 10 folds in the supplementary information, for parental and drug-adapted cancer cell lines, respectively. This might indicate that drug-adapted cancer cell lines develop a common resistance mechanism which makes them look more similar and more difficult for the model to differentiate them as they develop some common features as a results of drug treatment and development of resistance mechanism compared to parental cancer cell lines.

**Table 4:** Four class authentication.

Cell type	Mean F1 score	Standard deviation
Parental cancer cell lines	0.96	0.02
Drug treated cancer cell lines	0.91	0.03

#### Eight-class authentication task

On the combined data set classification problem (eight-class task), which is a more complex authentication task as it combines both four classes of parental and drug-treated cancer cell lines, an average of 0.91 F1-score is obtained with a standard deviation of 0.03, as shown in Table 5 below. A mean Area Under the Curve (AUC) of 0.95 was obtained with a standard deviation of 0.01 across the tenfold. Figure S7 and S10 in the supplementary information shows the ROC curves and the respective AUC for each class and the confusion matrices for the combined cancer cell lines, respectively. In Fig. S10 of the confusion matrices for the combined dataset, it can be noticed that the confusion occurred mostly between the parental class and its respective subline in most of the folds. This is due to the same genetic origin of the cell lines. The differences are only caused by the drug adaptation process.

**Table 5:** Eight classes authentication.

Cell type	Mean F1 score	Standard deviation
Combined cancer cell lines	0.91	0.03

#### Two-class authentication task

Two class authentications were also performed to see how the model performs in classifying between the parental cell lines and their drug-adapted sublines (Table 6). Although there are common features between the parental cell lines and the respective drug-adapted sublines as they come from the same genetic origin, our model managed to authenticate them with an average F1-score of around 0.95. Many mechanisms contributing to the development of drug resistance are at the biochemical level. The changes in biochemical properties of a cell can be visualised under a microscope^65^, which might be a reason why our model was able to differentiate between the parental and the drug-treated cancer cells.

**Table 6:** Two classes authentication.

Cell line	Average F1 score	Standard deviation
COLO-704/ COLO-704rCDDP1000	0.90	0.01
EFO-21/ EFO-21rCDDP2000	0.94	0.02
EFO-27/ EFO-27rCDDP2000	0.98	0.03
UKF-NB-3/ UKF-NB-3rOXALI4000	0.98	0.03
Average	0.95	0.02

The promising results of our method means that it may be used for authentication of cancer cell lines in conjunction with STR or for tasks where STR cannot be utilised.

### Effect of sample size

Table 7 shows the performance of our optimal model when trained with different training sample sizes. It is crucial to know the optimum number of training samples sufficient for a deep learning model to generalise, especially with biomedical images as it is usually difficult to get large data samples for training. For this investigation, we kept the test sample sizes constant for fair comparison while reducing the training sample size stepwise by 20%, from 100 to 20%. Table 7 shows that a drop from 100 to 80% in the number of training images from the original number of training image samples (i.e. from 1566 to 1253 training sample images) had a small impact on the F1-score, with a drop of 3%. Further reduction of the training data set has a significant negative effect on the model, with a significant reduction of the F1-score and an increase in the standard deviation. This suggests that, together with transfer learning and data augmentation techniques, a larger training data sample size will be required for better results.

**Table 7:** 10 folds cross validation with training sample size drop.

Percentage (%) of training sample size	Mean F1-score	Standard deviation
100	0.91	0.03
80	0.88	0.04
60	0.80	0.12
40	0.72	0.13
20	0.57	0.16

### Per-class performance

When training a machine learning model, the model can learn to classify some classes well and fail on other classes of the same problem. This might be due to the quality of the data in a particular class, imbalances between the datasets or a lack of diversity among the training samples in a particular class that prevents the model from capturing the patterns. It is important, therefore, to see how the trained models perform in classifying each class separately. The model performance in authenticating each class was studied and reported in Table 8. These results were extracted from the classification report of the eight-class authentication task. The average F1-score of each class across the ten folds and its standard deviation was calculated and reported in Table 8. The model had low confidence in authenticating the cancer cell lines EFO-21^r^CDDP^2000^ (mean F1-score 0.76) and EFO-27^r^CDDP^2000^ (mean F1-score 0.71), with the highest standard deviations out of all other cell lines. Further analysis on this case is presented later in the paper to find out the reason of this case.

**Table 8:** Model performance per class.

Class	Mean F1-score	Standard deviation
COLO-704rCDDP1000	0.95	0.03
COLO-704	0.96	0.03
EFO-21rCDDP2000	0.76	0.12
EFO-21	0.95	0.04
EFO-27rCDDP2000	0.71	0.16
EFO-27	0.95	0.06
UKF-NB-3rOXALI4000	0.98	0.04
UKF-NB-3	0.99	0.03

### Model confidence

It is important to understand how confident a trained model is in predicting the unseen dataset. Low confidence when making a correct prediction or high confidence when making an incorrect prediction may indicate the low performance of a model while high confidence in correct predictions and low confidence in wrong confidence may indicate the high performance of a model.

The probabilistic confidence of the model when making correct predictions was compared to when incorrect predictions were made (Table 9). This was done by randomly picking one image sample from the training sample set of each class. Overall, our model was very confident in predicting the correct class by attaining an average probability confidence of 0.92 for true positive predictions compared to 0.64 for false positive predictions. The lowest confidence of 0.76 and 0.78 were observed when correctly identifying EFO-27^r^CDDP^2000^ and EFO-21^r^CDDP^2000^, respectively. We conducted a further investigation on the EFO-21 and EFO-27, both parental and drug treated cancer cell lines to find out the reasons of the low model performance on the two cell lines, as reported in “Further investigation on EFO-21 and EFO-27” section. The model had the highest confidence in classifying COLO-704 and UKF-NB-3 cells with confidence of 0.99 and 1.0 probabilities respectively. The model had a low average confidence of 0.64 when predicting a wrong class. The high confidence of the model when making correct predictions and the low confidence when making false prediction mean that, our trained model can be trusted in correct authentication of cancer cells.

**Table 9:** Model confidence.

Cell line	Correct confidence	Wrong confidence
COLO-704rCDDP1000	0.93	0.76
COLO-704	0.99	0.74
EFO-21rCDDP2000	0.78	0.70
EFO-21	0.96	0.73
EFO-27rCDDP2000	0.76	0.75
EFO-27	0.96	0.79
UKF-NB-3rOXALI4000	0.97	0.68
UKF-NB-3	1.0	0
average	0.92	0.64

### Further investigation on EFO-21 and EFO-27

Due to the low performance in authentication of the drug-adapted sublines of EFO-21 and EFO-27 in the per-class performance of the optimal model (Table 8) and also in the model confidence investigation (Table 9), a further investigation was conducted on these cell lines. This was done by testing the F1-score values by which the model can discriminate between the parental EFO-21 and EFO-27 cell lines and between their drug-adapted sublines (Table 10).

**Table 10:** Investigation on Efo-21 and Efo-27.

Cell line	Mean F1-score	Standard deviation
Efo-21/Efo-27 Parental	0.94	0.01
Efo-21/Efo-27 Drug treated	0.60	0.05

The results show that our optimal model performed well in the authentication of the parental cancer cell lines, but was less reliable in the authentication of the cisplatin-resistant sublines (Table 10). These results are also supported by the performance observed in the confusion matrices of the parental cancer cell lines of EFO-21 and EFO-27 (Fig. S8 in the supplementary information), and of their drug-adapted sublines (Fig. S9 supplementary information). This may suggest that, apart from the drug-adapted cancer cell lines may be developing the same resistance mechanism which becomes harder for a deep learning model to authenticate as seen in Table 4 above, the parental cancer cell lines treated with the same anti-cancer drug develops more similar resistance mechanism compared to those treated with different anti-cancer drug.

## Discussion

The common issue of misidentification of cancer cell lines requires new ways for performing cell line authentication in a laboratory environment. Current authentication methods are expensive, time-consuming and cannot differentiate between cell lines of the same genetic origin, like parental cancer cell lines and their drug-adapted sublines. Resistance formation is associated with morphological changes that make drug-adapted cell lines distinguishable from the parental cell line. Hence, approaches using computer-aided digital image analysis can be used to develop effective authentication approaches that can be easily included into the daily laboratory routine and which may complement, to assist other authentication methods. Such methods may also enable the discrimination between cell lines of the same genetic origin, a task for which established methods are currently lacking. Such approaches have the potential to improve the reliability of research results due to the reduction of the use of misidentified cell lines^4–7^. By using deep learning, we have demonstrated that, it is possible to authenticate cancer cell lines, including parental cancer cell lines and their drug-adapted sublines, based on image recognition. Our data also suggest that resistance formation to a certain drug may be associated with specific morphological changes. An improved understanding of such processes may enable the further development of image-based strategies to gain mechanistic insights. Furthermore, our results suggest that, apart from the drug-adapted cancer cell lines developing the same resistance mechanism, cancer cell lines treated with the same drug develop much similar resistance mechanisms compared to those treated with different drugs, as it was demonstrated in the studying of the trained model confidence. Hence, our results are not only promising with regard to the development of novel cell line authentication approaches but also provide initial evidence that image-based methodologies can be developed as tools for the performance of functional and mechanistic studies. This is a proof of concept that, image-based methods with deep learning can be used to assist the current existing state of the art authentication methods like the STR.

## Conclusion

To this end, this thesis has successfully demonstrated the application of CNN in the authentication of cancer cell lines. Based on the pilot classification task result, techniques like transfer learning and data augmentation has significantly improved the model’s performance. Furthermore, a multi-stage transfer learning has shown a significant increase of 4% in F1-score in another study by Ravi et al.^45^ because they used a dataset from their domain in the intermediate stage, compared to a 1% increase in F1-score in our case, this is because we were limited with the unavailability of publicly available cancer cell lines to be used in the intermediate stage. This means researchers should look for datasets from the domain of their target task when in need of applying multistage transfer learning. Finally, studying of a model confidence might be used as a way for detecting the prediction uncertainties of a trained model as it has been demonstrated in this paper.

## Supplementary information

Supplementary Information.
