# Supplementary material for: Towards image-based cancer cell lines authentication using deep neural networks
Source: Sci Rep. 2020 Nov 16;10:19857. doi: 10.1038/s41598-020-76670-6 (PMC7670423; doi:10.1038/s41598-020-76670-6)
Supplement: Supplementary file 1 — Supplementary Information. [file 41598_2020_76670_MOESM1_ESM.pdf]

Towards Image-based Cancer Cell Lines Authentication Using Deep Neural Networks

Deogratias Mzurikwao, Muhammad Usman Khan, Oluwarotimi Williams Samuel, Jindrich Cinatl jr, Mark Wass, Martin Michaelis, Gianluca Marcelli, Chee Siang Ang

Supplementary material.

Our models were trained on a Tesla GPU server available at The University of Kent with the following specifications

a) HPE ProLiant DL380 Gen9 Server Chassis
b) 2x Intel Xeon E5-2667v4, 3.2GHz, 8 core CPUs
c) 2x NVidia Tesla K80 GPU/Compute Cards (~10k GPU cores)
d) 192GB, DDR4 2400 RAM
e) 2x 480GB SSDs in RAID 1 (System Data/Applications)
f) 2x 8Gb/s Fibre Channel to HDS External Enterprise SAN Storage (User Code/Datasets)
g) RedHat Enterprise Linux 7

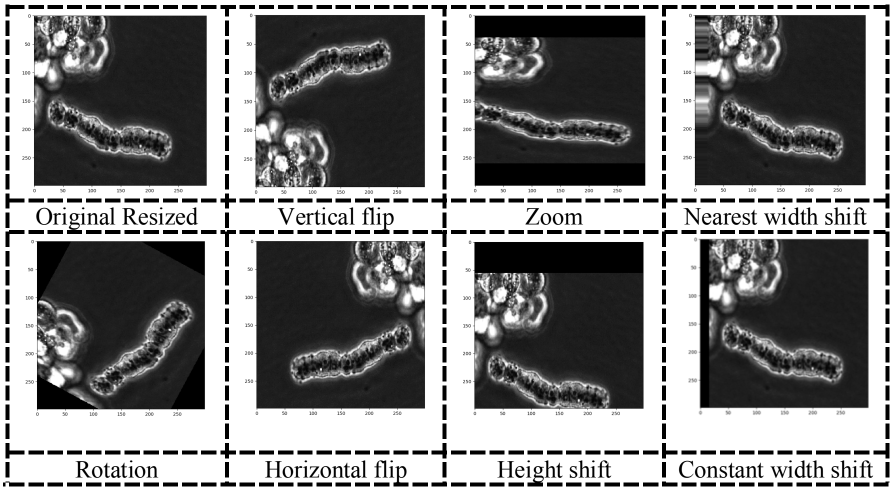

Figure S1: Image augmentation samples with different twisting parameters with nearest and constant width shifts pixels assignments.

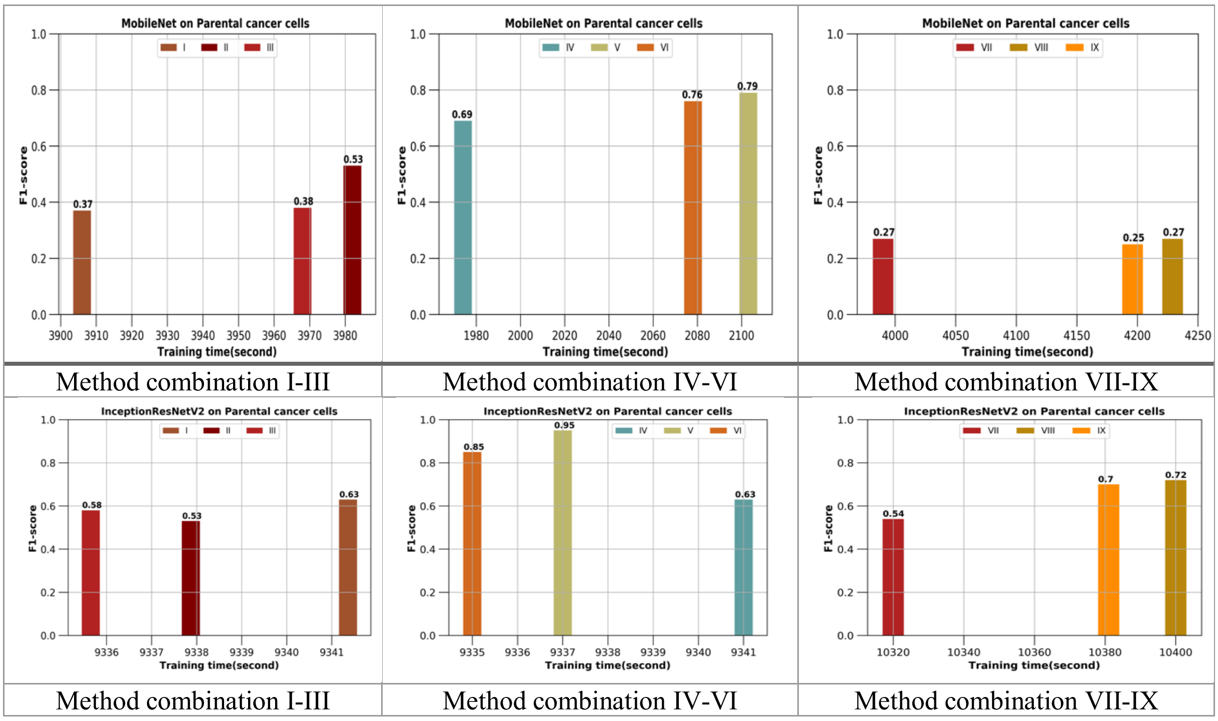

Figure S2: Parental cancer classification model performance under different methods combination, Top row for MobileNet and lower row for InceptionResNet V2, each block shows performance of the three tested
[illegible]

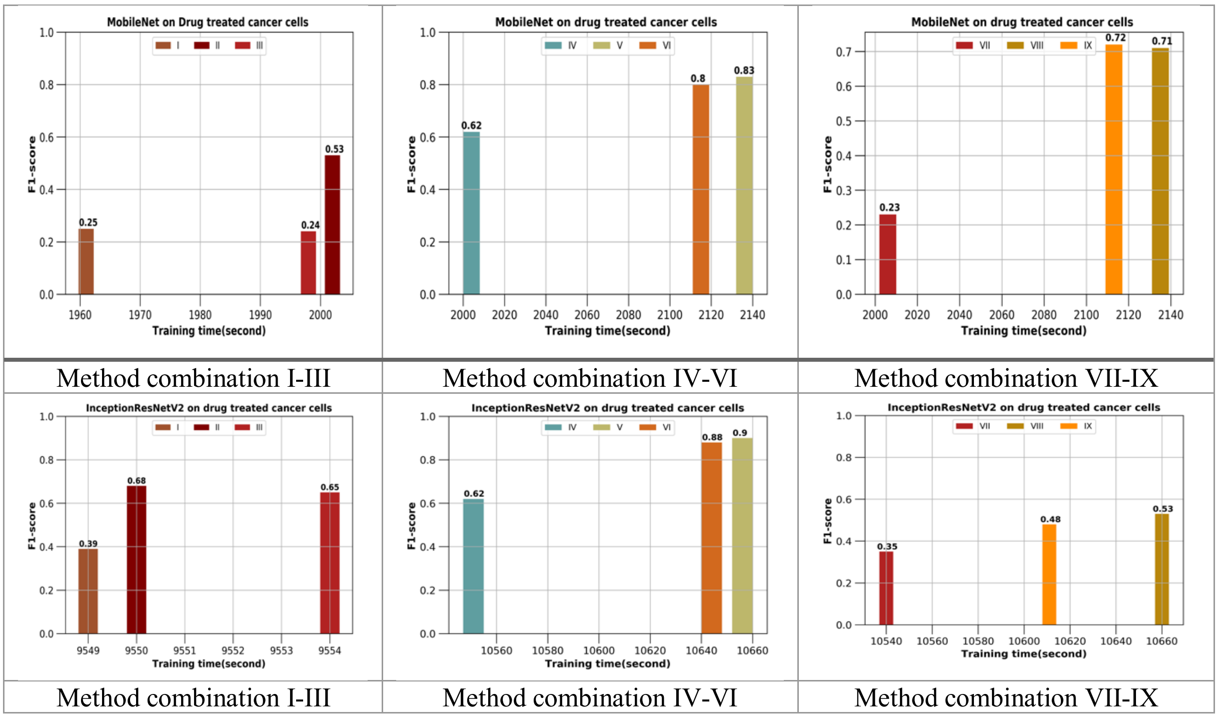

Figure S3: Drug treated cancer classification model performance. Top row for MobileNet and lower row for InceptionResNet V2, each block shows performance of the three tested combinations.

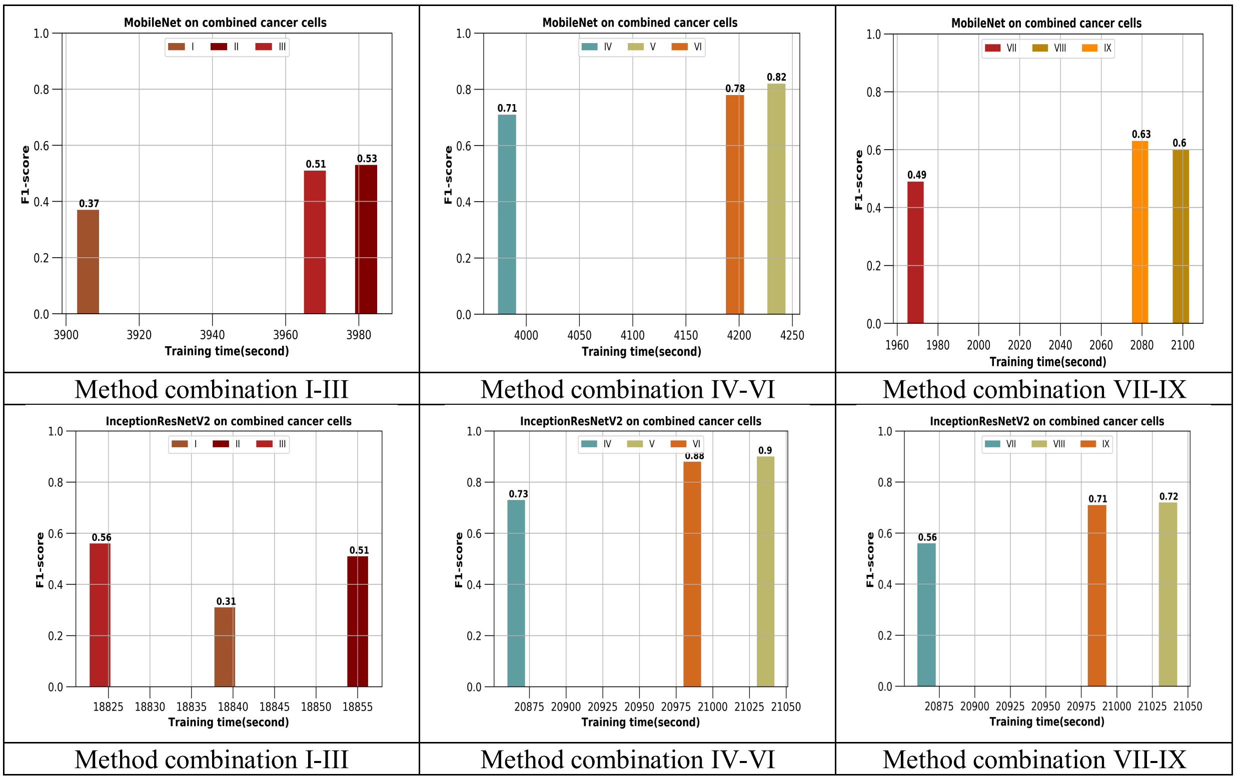

Figure S4: combined cancer classification model performance, Top row for MobileNet and lower row for InceptionResNet V2, each block shows performance of the three tested combinations

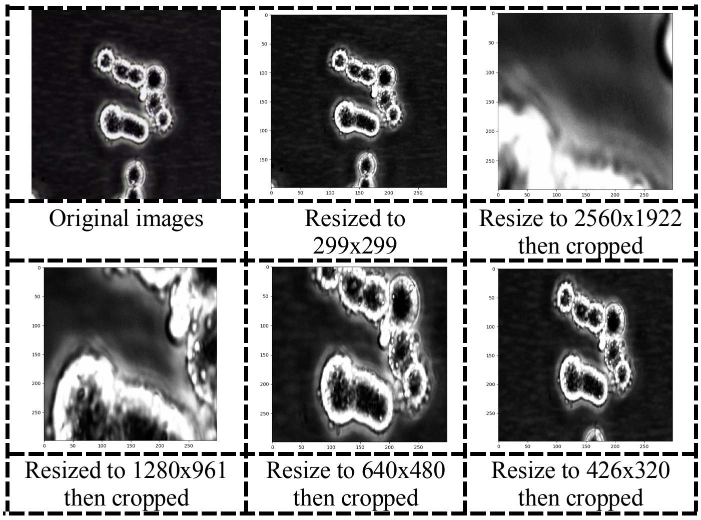

Figure S5. Resizing and cropping

Table S1. Resizing and cropping

Model	Test F1-score
Resize to 2560x1922 then crop	0.44
Resize to 1280x961 then crop	0.62
Resize to 640x480 then crop	0.78
Resize to 426x320 then crop	0.84
Resize to 299x299	0.87

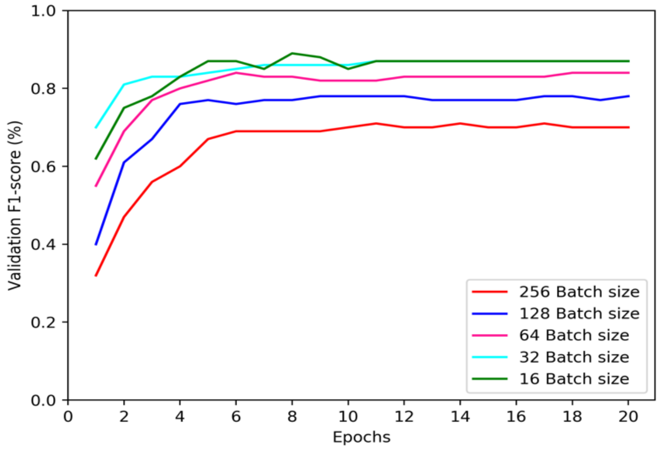

Figure S6. Batch size learning curves

Table S2: Batch size f1-score

Batch Size	Test F1-score	Training accuracy	Training F1-score	Train Time per Image
16	0.87	0.9463	0.97	13.49
32	0.87	0.9355	0.96	13.04
64	0.84	0.9185	0.94	12.62
128	0.77	0.8937	0.91	12.42
256	0.68	0.7885	0.80	12.79

Table S3. Different architecture

Fully Connected Layers	Test F1-score	Training accuracy	Training F1-score
1x 256 Neurons	0.83	0.9406	0.96
1x512 Neurons	0.86	0.9432	0.97
1x1024 Neurons	0.87	0.9463	0.97
2x1024 Neurons	0.87	0.9296	0.96

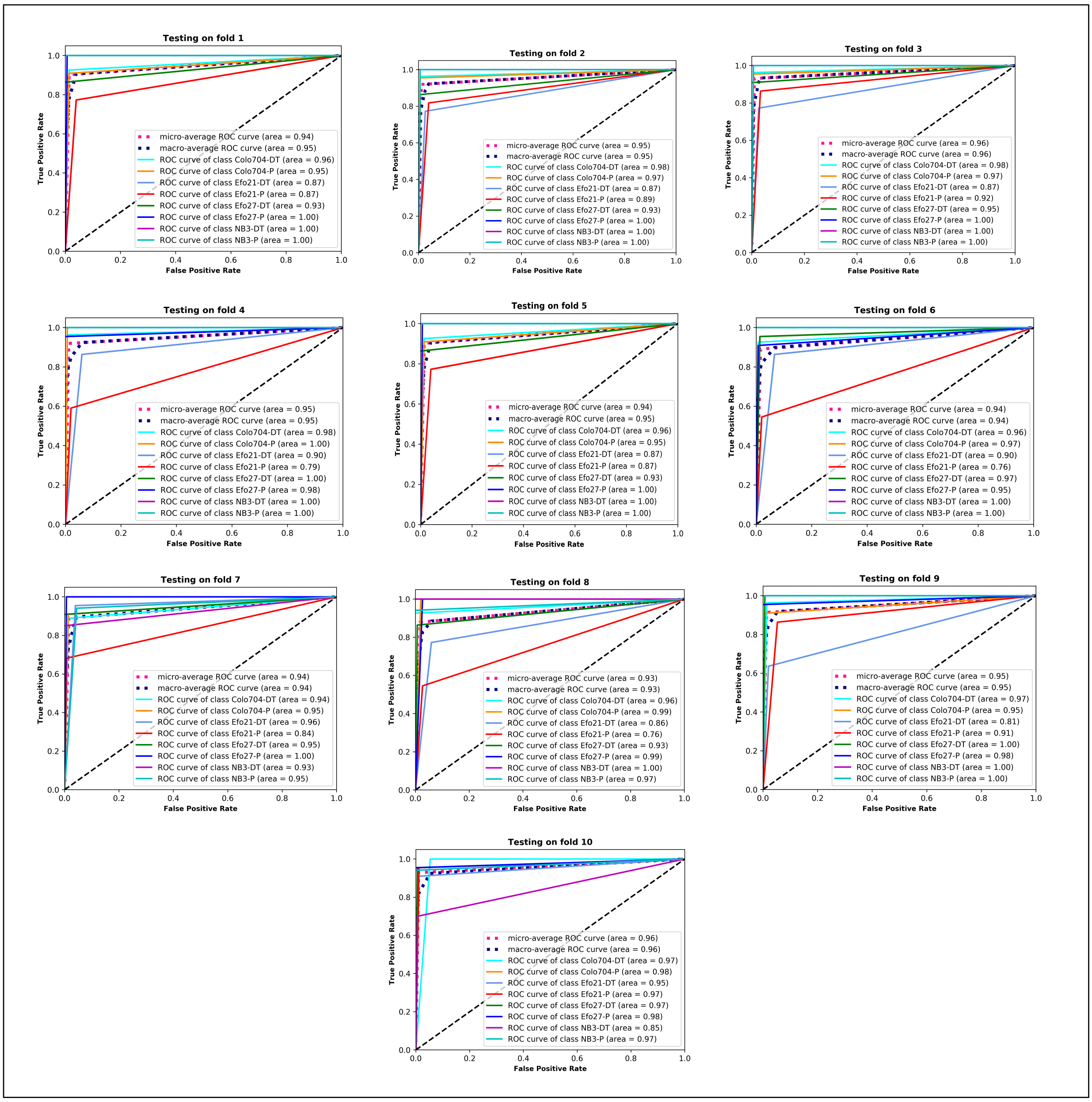

Figure S7: ROC curves of combined cancer cell lines. For clear visualisation of the Figure, Parental cancer cell lines have been abbreviated as P and DT for Drug treated cancer cell lines.

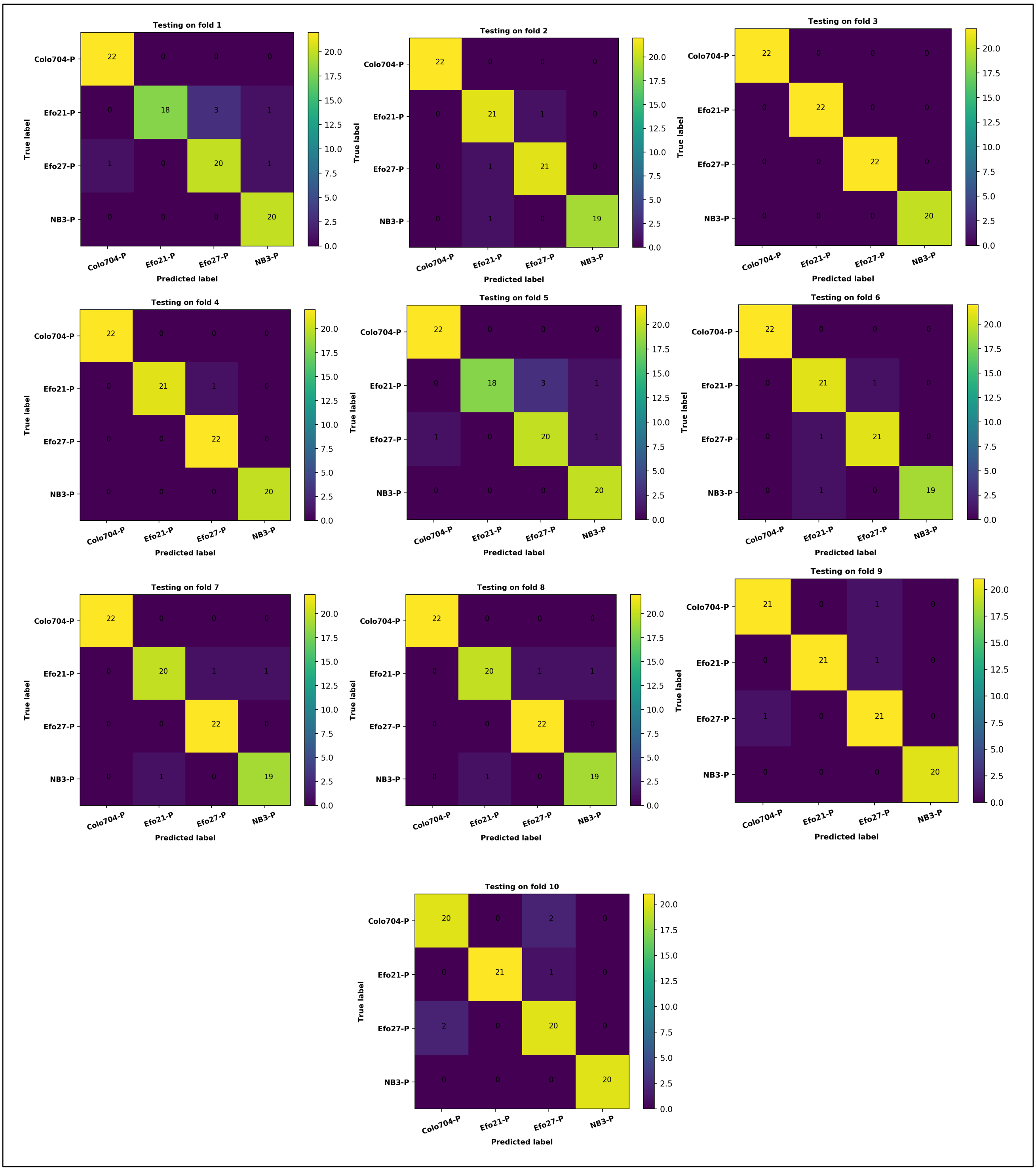

Figure S8: Confusion matrices of parental cancer cell lines. For clear visualisation of the Figure, parental cancer cell lines have been abbreviated as P.

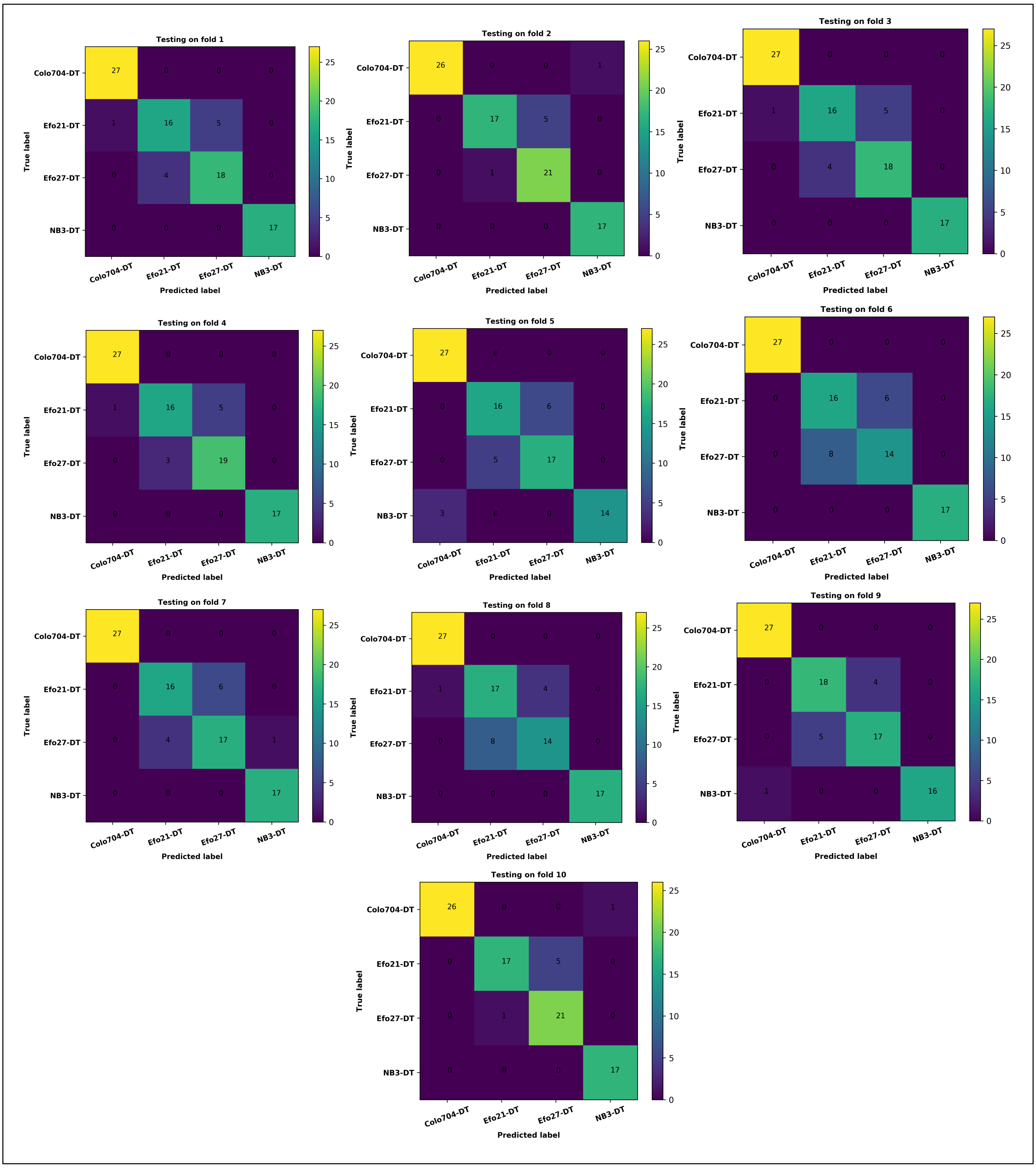

Figure S9: Confusion matrices of drug treated cancer cell lines. For clear visualisation of the Figure, Drug treated cancer cell lines have been abbreviated as DT.

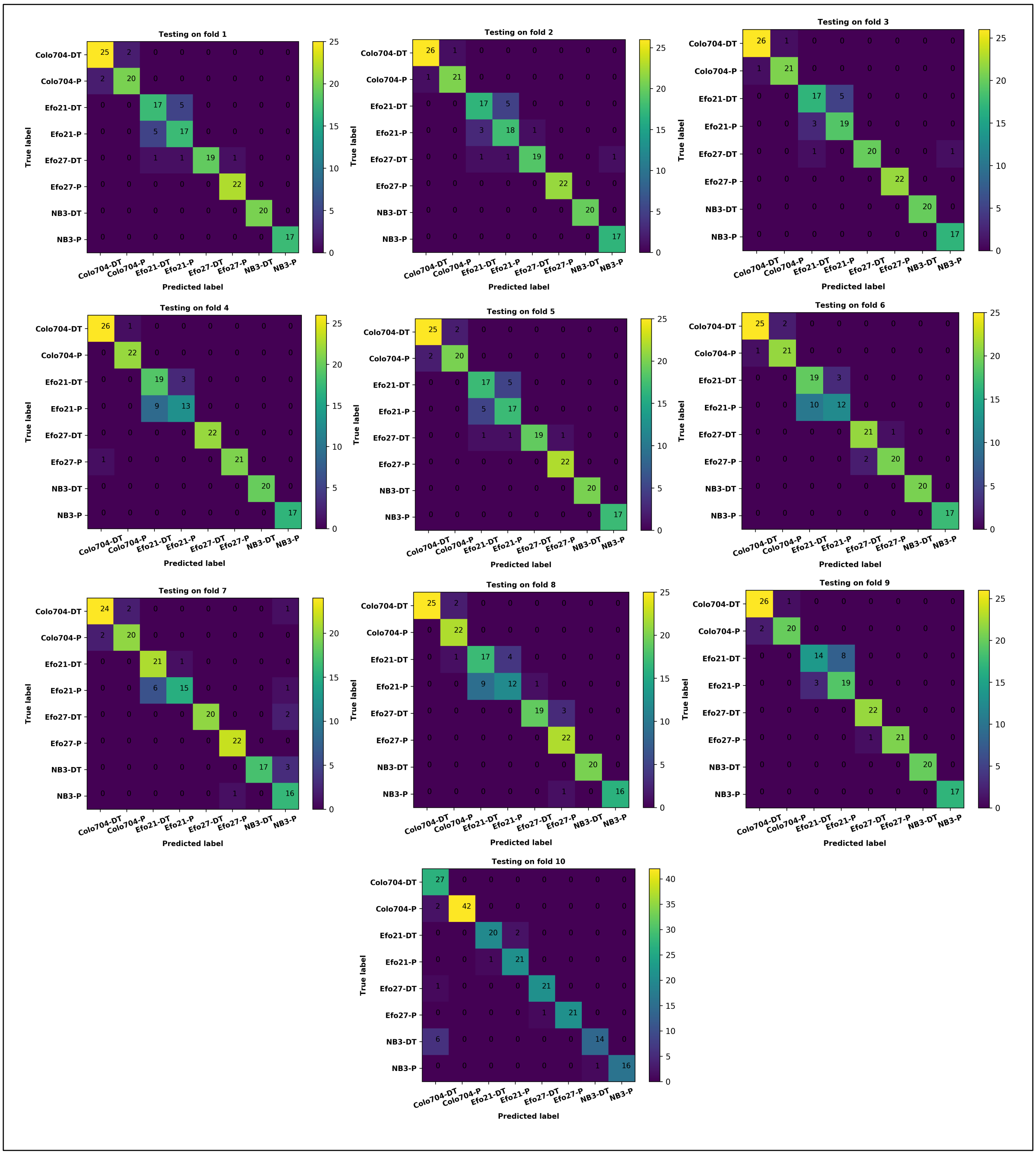

Figure S10: Confusion matrices of combined cancer cell lines.
